# Eating in the Absence of Hunger in Hispanic Preschoolers: Relationships with Child Sex, Food Preference, and Weight Status

**DOI:** 10.3390/nu17071266

**Published:** 2025-04-04

**Authors:** Adriana Verdezoto Alvarado, Bin C. Suh, Michael Todd, Jacob Szeszulski, Elizabeth Lorenzo, Meg Bruening, Clare Schuchardt, Rebecca E. Lee

**Affiliations:** 1 Department of Behavioral Health and Nutrition, College of Health Sciences, University of Delaware, 100 Discovery Blvd., Newark, DE 19713, USA; averdezo@udel.edu; 2 Bureau of Assessment and Evaluation, Arizona Department of Health Services, 150 N 18th Ave, Phoenix, AZ 85007, USA; bin.suh@azdhs.gov; 3 Edson College of Nursing and Health Innovation, Arizona State University, 550 N 3rd Street, Phoenix, AZ 85004, USA; mike.todd@asu.edu; 4 Institute for Advancing Health Through Agriculture (IHA) at Texas A&M AgriLife Research, 17360 Coit Rd, Dallas, TX 75252, USA; 5 School of Nursing, University of Texas Medical Branch, 301 University Blvd., Galveston, TX 77555, USA; ellorenz@utmb.edu; 6 Department of Nutritional Sciences, College of Health and Human Development, Pennsylvania State University, 110 Chandlee Lab, University Park, PA 16802, USA; mmb203@psu.edu; 7 Center for Health Promotion and Disease Prevention, Edson College of Nursing and Health Innovation, Arizona State University, 550 N 3rd Street, Phoenix, AZ 85004, USA; cschucha@asu.edu

**Keywords:** eating in the absence of hunger, preschool children, Hispanic children, weight status

## Abstract

**Background/Objectives:** This study examines the relationship of eating in the absence of hunger (EAH) with child sex, food preference, and body mass index (BMI) percentiles in primarily Hispanic preschoolers, an understudied population. **Methods:** This was a secondary analysis of data from 211 children (79% Hispanic) aged 3 to 5 years from low-income families who completed a cluster randomized controlled trial from September 2017 to June 2020. Weight and height were used to calculate BMI percentiles. Sweet (animal crackers) and salty (pretzels) snacks were used to conduct a validated classroom-based EAH assessment. A generalized estimating equation (GEE) approach investigated associations between the grams of snacks consumed and BMI percentiles. A set of nested multivariable GEEs were estimated, while adjusting for potentially important covariates. **Results:** Boys significantly consumed more snacks than girls (13.34 ± 9.71 g vs. 8.13 ± 7.36 g; *p* < 0.001). Children who indicated greater preference for sweet snacks consumed more sweet snacks (*r* = 0.19; *b* = 2.05, *p* < 0.001) and total grams of total snacks (*r* = 0.18; b = 2.42, *p* = 0.004) but not salty snacks (pretzels). Consuming more sweet snacks was significantly associated with higher BMI percentiles (*b* = 0.55; *p* = 0.024). **Conclusions:** The findings suggest that a preference for sweet snacks is associated with EAH, and eating sweet snacks in the absence of hunger is related to higher BMI percentiles. Obesity prevention programs may focus on addressing eating sweet snacks in the absence of hunger starting in early childhood.

## 1. Introduction

Eating in the absence of hunger (EAH) has been associated with increased weight, high body mass index (BMI), and increased adiposity among children [1,2,3,4,5,6,7,8,9]. EAH is defined as eating when not hungry or past satiety [3]. EAH is characterized by an inability to self-regulate satiety and dietary intake and has been used to measure disinhibited eating among children [10,11,12]. Learning healthy eating habits through better self-regulated calorie consumption at a young age may prevent caloric surplus and developing overweight or obesity [11,13,14,15]. Despite a growth of literature in this arena, there has been less focus on younger children (3–5 years) and Hispanic populations, who exhibit higher prevalence of overweight and obesity [16].

The assessment of EAH was developed under controlled laboratory settings [3], with less work in natural settings. Although internal cues, such as taste preferences stemming from adrenocortical regulation [17,18], and cognitive development [11,19,20] can influence EAH, a wide variety of external, context-specific cues may also contribute to this phenomenon. For example, feeding practices at home or school [15,21,22] and role modeling [23] along with practices such as the unrestricted snacking of preferred foods and serving large portion sizes can influence EAH [24,25,26,27,28,29,30,31]. Observing children in naturalistic settings, such as early care and education (ECE) facilities (e.g., preschools), can be advantageous for informing future research, policy, and practice, since children spend most of their time during weekdays at some kind of non-parental care [32,33].

Although EAH can be influenced by both internal and external factors, there is a need to investigate the relationship between food preferences (sweet vs. salty) and EAH. Humans are born with a preference for sweet flavors and are more likely to prefer sweet foods compared with other flavors by the age of five [34]. Exposure to sweet foods in early childhood may heighten this preference, potentially increasing the risk of developing obesogenic behaviors later in childhood. The consumption of highly palatable, ultra-processed foods, which are often very sweet (e.g., candy), is associated with weight gain in childhood [35]. One study reported that overall EAH and, in particular, the over consumption of sweets predicted fat gains in middle childhood [2]. Salty foods can also be highly processed and calorie dense and may also contribute to childhood obesity, but fewer studies have investigated this pathway. Little research has investigated eating these foods in the absence of hunger in ECE facilities.

EAH may also be affected by child sex [1,3,13,14]; however, studies have reported conflicting findings. For example, Fisher and Birch [3] suggested that girls’ perception and awareness of food availability might be higher than boys’, because girls have a better understanding of what their parents offered and restricted within the household. Greater awareness of food availability could result in higher caloric intake for girls during EAH assessments [14]. In contrast, in a study of low-income 2- to 3-year olds [1] and another of Australian 3- to 5-year olds [36], boys were more likely to consume more calories during EAH assessments than girls. One hypothesized reason that boys may have consumed more calories in EAH assessments is that they have less self-control toward food and wait less time to consume greater amounts of EAH snacks than girls [13]. Other authors have posited that mothers’ may differentially pressure boys to eat more than girls [21,37]. To date, there is not a clear understanding of how child sex affects EAH.

Although previous research has explored the relationship between EAH and obesity, these studies have been carried out primarily in laboratory settings with small sample sizes [11,14,21]. There is not a clear understanding of whether child sex influences EAH, how food or taste preferences influence EAH, and how these relationships function in younger children [1,36]. Finally, there are few investigations in primarily Hispanic children, at a high risk of developing obesity [16]. The purpose of this study was to examine EAH and BMI percentiles of a sample of primarily Hispanic children who were enrolled in ECE facilities that participated in the Children and Adult Care Food Program (CACFP) [38]. Our research aimed to compare the associations of child sex, food preferences for sweet or salty snacks, and BMI percentile during an adapted EAH test for use in ECE facilities [12]. We hypothesized that (1) boys would consume a greater amount of snacks than girls, (2) food preference would result in greater consumption of snacks, and (3) greater snack consumption after a standardized EAH test would be positively associated with BMI percentiles.

## 2. Methods

### 2.1. Participant Recruitment

This study was a secondary data analysis using baseline data from an ongoing cluster randomized controlled trial of Sustainability via Active Garden Education (SAGE), a garden-based, multilevel strategy to increase physical activity and improve dietary habits in preschool-aged children [39]. As previously published, SAGE used a 12-session ECE facility-based intervention for children aged 3 to 5 years old in the Phoenix metropolitan area. In the city of Phoenix, 42.7% of the population is Hispanic or Latino [40]. Children attending ECE facilities with census tracts with >30% Hispanic population were intentionally recruited for the study [39]. Additional eligibility criteria included ECE facilities participating in CACFP or the National School Lunch program; being located in census tracts of at least 3000 residents; being a full-day school where children could attend 4 or 5 days a week; and having a drop-off/pickup window fewer than 60 min. Family home care sites were excluded from participation [39]. One classroom per ECE facility was enrolled in the study. A total of 26 ECE facilities participated in 3 cohorts between September 2017 and June 2020. Each classroom had approximately 18 to 25 children, and SAGE enrollment was 50% to 75% of children per classroom. Since all children in the classroom were invited to participate, researchers did not exclude based on race/ethnicity; hence, the sample is of mainly Hispanic children. Parents provided written informed consent in their preferred language (English or Spanish) to enroll their child in SAGE. All children provided verbal assent at the time of assessment. This study was approved by the Institutional Review Board at Arizona State University (STUDY00003761) and is registered at ClinicalTrials.gov (Identifier: NCT 03261492).

An initial sample of 272 children was eligible to participate in EAH assessment. Participants who did not complete demographic variables (*n* = 14), anthropometric measures (*n* = 33), and/or the EAH assessment (*n* = 39) were excluded from analysis. This resulted in a final sample of 211 children.

### 2.2. Sociodemographic Characteristics

Demographic information, including child’s sex (parent-selected: male/female), race (parent-selected from a list: White, Black/African American, American Indian or Alaskan Native, Asian, Native Hawaiian or other Pacific Islander, other [specify]), ethnicity (parent-selected from Hispanic origin [Mexican, Puerto Rican, Cuban, other] or non-Hispanic), and age, along with parent’s sex (self-selected: male/female), age, educational attainment, and household income were collected via questionnaires administered to parents during baseline. ECE facility was classified as Head Start facilities versus “other ECE facilities” not participating in Head Start.

### 2.3. EAH Assessment

EAH is an assessment of snacking behaviors in the absence of hunger that was developed by Fisher and Birch [3] and modified by Soltero and colleagues [12] to be used in ECE facilities. Assessments were conducted in classrooms immediately after the normally scheduled lunch. Children received one snack bag each of sweet snacks (animal crackers) and salty snacks (pretzels) labeled with the child’s name. Snack bags were pre-portioned to 30 g of each snack per bag, ensuring consistency across participants. Each snack bag was weighed twice in grams, averaged, and rounded to the nearest tenth; each bag weight was recorded under the specific child’s name and labeled. These snacks were in compliance with ECE facility regulations. Children were presented with posters of “yummy”, “just okay”, and “yucky” faces to identify food preference or “liking” and were instructed to taste one piece of each snack at a time. Their food preference response was documented. Following the tasting, children were provided coloring sheets and crayons and instructed to color and/or eat ad lib. Children were told that they may eat the snacks or play with the coloring sheets while the teacher prepared for the next activity. If any children shared food with another participant, the research assistants instructed children to only eat their own snack, and the snacks were returned to the child’s bag that shared the snack at the end of the assessment. If any children dropped food on the ground, it was also added to the specific child’s bag at the end of the assessment. Research assistants documented any sharing or dropping of snacks on the data collection forms. Research assistants set a 10 min timer, after which the children were instructed to stop eating, and all snacks were collected. Again, the sweet (animal crackers) and salty (pretzels) snacks were each weighed to the nearest 0.1 g 2 times, and the 2 weights were averaged. The post-activity average weight for each bag (the weight of the remaining animal crackers or pretzels) was then subtracted from its pre-activity average weight, yielding an amount of each snack consumed. Differences < 0 g were scored as 0 g (i.e., none of the snack was consumed). With the exception of one difference of −3.7 g, these negative difference values were >−0.8 g. Because sweet and salty snacks had similar energy content (120 calories for 30 g of animal crackers, 128 calories for 30 g of pretzels), the amounts of sweet and salty snacks consumed were summed to yield a total amount of snacks consumed.

### 2.4. Anthropometric Measurements

Anthropometric measurements were measured twice by trained research staff to enhance accuracy. Children were asked to remove their shoes. Weight was obtained using a Tanita body composition analyzer (model TBF-310, Tanita Corporation, Tokyo, Japan) to the nearest half pound. Height was measured with a portable Seca stadiometer (model 213, Seca Corporation, Hamburg, Germany) to the nearest quarter inch. All measures were averaged. Age- and gender-normed BMI percentiles were calculated based on CDC growth charts [41]. BMI percentile values were used to classify children as normal weight (5th to <85th percentile), overweight (85th to <95th percentile), and obese (≥95th percentile). Only 2 children were classified as underweight (<5th percentile) and were included in the normal weight category.

### 2.5. Statistical Analyses

Because the data analyzed had an inherently multilevel structure (children and caregivers nested within 26 ECE facilities), we first estimated intraclass correlation coefficients (ICCs) to examine the degree of clustering (non-independence) in snack consumption and children’s BMI percentile values measured at the ECE facility level. Because non-trivial clustering was observed for snack consumption (ICCs = 0.144, 0.000, and 0.174 for sweet snacks consumed, salty snacks consumed, and total snacks consumption, respectively) and BMI percentile (ICC = 0.045), data were analyzed using a generalized estimating equation (GEE) approach. For the analyses reported below, all observations (*n* = 211) were used. Parallel analyses excluding the observation with a large negative pretzel consumption value (−3.7 g) were also performed. The results of those analyses, which were not substantively different, are not reported here but are available from the authors upon request.

First, bivariate associations between key variables were examined to test Hypothesis 1 concerning the relationship between child’s sex (independent variable) and snack consumption (dependent variable) and Hypothesis 2 concerning food preference (independent variable) and snack consumption (dependent variable). The associations of EAH snack consumption variables (amount of sweet snack, amount of salty snack, and total amount of snacks) with food preference items and BMI percentile were described using Pearson’s correlation coefficients and then tested using single-covariate GEE models with snack consumption as the outcome variable. Similarly, the associations of EAH snack consumption with child sex, racial/ethnic group, ECE facility type, and weight status were examined using group means and then tested in single-covariate GEE models.

Next, we estimated a hierarchical set of multivariable GEEs to examine associations of EAH variables and food preferences (independent variables) with BMI percentile (dependent variable), while adjusting for potentially important background covariates. To test our first hypothesis, Model 1 included child sex, race/ethnicity, and ECE facility type (Head Start vs. “other childcare facility” [reference category for analyses]) as covariates. To test our second hypothesis, Model 2 included all Model 1 covariates plus the food preference rating for each type of snack as covariates. The final model (Model 3) tested our third hypothesis, that greater snack consumption (independent variable) would be associated with higher BMI percentile (dependent variable), and it included all Model 2 covariates plus the amount of sweet snacks (animal crackers) consumed and the amount of salty snacks (pretzels) consumed as covariates. All GEEs specified Gaussian (normally distributed) errors and an exchangeable working correlation structure. GEE coefficients are represented by “*b*” in the text and tables below.

Univariate statistics were computed in the Statistical Package for the Social Sciences (SPSS) version 26 (IBM Corp., Armonk, NY, USA). ICCs were calculated using variance component estimates from the minque package [42], and GEE models were estimated using the geepack package [43], both in R version 4.2.2 [44]. Statistical significance was set at *α =* 0.05.

## 3. Results

### 3.1. Sample Characteristics

Half (*n* = 109, 52%) of the children were female. Over two-thirds (72%) were normal weight, 13% were overweight, and 15% of children had obesity. Ages ranged from 39 to 59 months with a mean of 51.5 months (4.3 years). Most children (68%) were Hispanic. Nearly half (*n* = 96; 46%) of the children were enrolled in Head Start facilities. Most parents/caregivers (89%) in the sample were female, and nearly half (46%) of the 150 in the sample who responded to the item reported a household income of USD 24,000 or less per year. Of those parents who responded to items regarding their child’s race (*n* = 207), 93 (45%) identified as White, 32 (16%) as Black or African American, 4 (2%) as American Indian or Alaska Native, 2 (1%) as Pacific Islander, 42 (20%) as another race, 9 (4%) as more than one race, and 25 (12%) said they did not know or were not sure. Of the 199 who responded regarding Hispanic/Latino ethnicity, 138 (69%) identified as Hispanic or Latino/a, 60 (30%) identified as non-Hispanic, and one did not know or was not sure. Additional descriptive information is presented in Table 1.

### 3.2. Preliminary Bivariate Analyses

Boys consumed more grams of sweet snacks (*b* = 2.39, *p* = 0.015), salty snacks (*b* = 2.09, *p* < 0.001), and total snacks (*b* = 4.26, *p* < 0.001) than girls (see Table 2 for group *M* and *SD* values). Preference for sweet snacks was positively associated with the grams of sweet snacks (*r* = 0.17; b = 1.75, *p* < 0.001) and total grams of snacks consumed (*r* = 0.14; b = 1.64, *p* = 0.027), but not grams of salty snacks consumed (*r* = −0.00, *b* = −0.02, *p* = 0.967). The total amount (grams) of snacks consumed was strongly positively related to the amounts of sweet snacks (*r* = 0.83) and salty snacks (*r* = 0.58) consumed. BMI percentile was significantly related to the grams of sweet snacks consumed (*r* = 0.13, *b* = 0.04, *p* = 0.013). Neither the grams of salty snacks consumed (*r* = −0.10; *b* = −0.02, *p* = 0.114) nor total grams of snacks consumed (*r* = 0.05, *b* = 0.02, *p* = 0.323) were significantly related to BMI percentile.

Sweet, salty, and total snack consumption did not differ significantly by ECE facility type (Head Start facilities vs. other facilities; *b* = −1.08, *p* = 0.442; *b* = −0.88, *p* = 0.170; and *b* = −2.13, *p* = 0.230, respectively) or by ethnicity (*b* = −1.72, *p* = 0.091; *b* = −1.41, *p* = 0.102; and *b* = −2.50, *p* = 0.093, respectively). Children within the normal weight percentile tended to consume smaller amounts of sweet snacks, salty snacks, and total snacks than either children with overweight percentiles (*b* = 0.30, *p* = 0.860; *b* = 0.24, *p* = 0.799; and *b* = 0.08, *p* = 0.970, respectively) or obese percentiles (*b* = 0.97, *p* = 0.531; *b* = −0.98, *p* = 0.306; and *b* = 0.51, *p* = 0.817, respectively), but these between-group differences were weak (0.00 < |Cohen’s d| < 0.15) and not significant.

### 3.3. Multivariable Analyses

Results from a hierarchical set of GEE models showed that the grams of sweet snacks consumed was generally the most important factor associated with BMI percentile (see Table 3). In Models 1 and 2, none of the covariates were significantly related to BMI percentile, and the fit of Model 2 was not significantly better than that of Model 1 (*X*^2^ (2) = 1.15, *p* = 0.562), indicating that no additional variability in BMI percentile was accounted for by food preference over and above that accounted for by background covariates. In Model 3, however, the amount of sweet snacks consumed was significantly and positively related to BMI percentile (*b* = 0.61, *p* = 0.026). Although salty snack consumption was not significantly related to BMI percentile (*b* = −0.57, *p* = 0.106), the fit of Model 3 was significantly better than that of Model 2 (*X*^2^ (2) = 11.02, *p* = 0.004), suggesting that jointly, higher sweet snack consumption and lower salty snack consumption accounted for significant variability in BMI percentile, adjusting for background covariates. Sweet snack consumption was the most compelling and consistent variable associated with BMI percentile.

## 4. Discussion

Understanding the associations of snacking practices and weight in preschool-aged children is important to guide efforts to prevent the development of overweight and obesity. The purpose of this study was to explore the relationships of child sex, food preferences, and BMI with EAH among primarily Hispanic preschoolers residing in Phoenix, Arizona. Consistent with our hypothesis and other studies [45,46,47], boys consumed more sweet, salty, and total amount of snacks than girls. Bivariable analyses found that children who indicated preference for sweet snacks consumed more sweet snacks and total snacks. Consuming sweet snacks during the EAH assessment was associated with higher BMI percentiles, regardless of food preferences. Overall, our findings highlight the importance of teaching preschool-aged children self-regulatory skills to prevent the temptation to overconsume snacks when hunger is not present.

Similar to other studies [1,48], we found a weak association of pretzel consumption with BMI percentiles; instead, there was a significant relationship between animal cracker consumption and BMI percentiles. This relationship became marginally significant after accounting for other independent variables, including a preference for sweet snacks, in the final regression model. Nevertheless, descriptive results showing a greater amount of sweet snack (i.e., animal crackers) consumption suggest that sweet snack preference could be a potentially salient driver of EAH and may be associated with children’s weight status. However, children’s eating behaviors could also be shaped by parental feeding styles, parental BMI, and the food environment [49]. In past research, preschoolers have reported greater enjoyment of foods containing higher amounts of added sugar [50] and were more prone to consuming sugary snacks during EAH [48,51]. Although the observed associations between sweet snack consumption and BMI percentile (*b* = 0.61) in this study were modest, increased added sugar intake during early childhood could contribute to excess weight gain. In this study, we used the standard (as stated on the packaging) serving size for animal crackers (the sweet snack) of 30 g. Following this study’s results, if the serving size were to be reduced by 10 g and consistently offered as a 20 g serving, then this reduction in snacking would be associated with approximately a 6% decrease in BMI percentile. In a similar vein, Wojcocki and Heyman [52] recommended preventing high sugar consumption by reducing consumption of 100% fruit juice in low-income ECE facilities (e.g., Head Start facilities). These findings underscore the importance of early interventions to promote healthier snack choices and self-regulation in young children, particularly when children present no appetite.

In Model 3, we found that there was a significant relationship between ECE facility type and BMI percentiles after controlling for other covariables. These differences could be attributed to variations in food and physical environments across ECE settings. Head Start facilities are federally funded and required to follow CACFP nutrition standards, which mandate offering components from the milk, fruit, vegetable, grain/bread, and meat/meat alternate food groups with meals, as well as providing structured physical activity opportunities for children [53]. In contrast, while some childcare facilities may participate in CACFP, they are not subject to the same level of standards. These structural differences in nutrition and physical activity could contribute to differences in children’s BMI percentiles. Given that both settings have room for improvement [54], future research should examine how differences in training, resources, and policies within ECE settings influence Hispanic children’s weight outcomes and their ability to develop self-regulation for energy intake, which plays a critical role in obesity risk.

Several limitations were identified in the current study. First, although EAH testing was carried out immediately following a meal, hunger was not reported during our assessment. Given that EAH is defined as eating in the *absence* of hunger, the lack of a hunger assessment is an important limitation. Assessing hunger could have explained why some children consumed greater amounts of snacks. However, assessing hunger among young children presents challenges, as they may have difficulty accurately reporting internal hunger cues [55,56]. Future research should incorporate validated physiological and behavioral markers (e.g., ghrelin levels) to strengthen the interpretations of EAH in preschool-aged children. In addition, longitudinal studies tracking snacking behaviors over time could provide insight into how early EAH patterns influence long-term self-regulation and obesity risk.

Another limitation is the potential influence of peer dynamics on reported food tasting preferences [57]; therefore, associations between snack consumption and food tasting preferences (“yummy”, “yucky”, and “just okay”) should be interpreted carefully. In a group setting, children may adjust their responses based on peer influence or social desirability rather than their actual taste preferences. For example, children may report liking or disliking a snack based on how their peers react rather than their true individual preference. Additional studies could explore strategies to minimize peer influence (e.g., private response methods) to obtain more accurate measures of children’s food preferences. In addition, offering children a limited variety of snacks could have affected food preferences or “liking” and altered consumption (e.g., animal crackers are presented with fun shapes, while pretzels may be less aesthetically appealing to children); however, previous investigations by this group have found that offering highly appetizing snacks may also bias results. For example, in a previous investigation by this group, children were offered M&Ms, and nearly all children consumed all of the snack, perhaps because of its high palatability or infrequent presentation in the home due to cost or other considerations [12]. Future research could explore how different snack types and presentation styles influence EAH behaviors across diverse populations.

Despite the limitations, there are several strengths in our study. SAGE is one of the first studies that collected EAH data in ECE classrooms. By assessing EAH in a naturalistic ECE setting rather than a laboratory, our study might provide a more ecologically valid representation of children’s real-world eating behaviors. Having teachers assist during assessments helped reduce error and improve data quality, enhancing the validity of our findings. In addition, the majority of our sample was Hispanic, an under-represented population in the literature. Additional studies are needed to determine how these findings can be applied to other populations. Future research could examine how children learn to regulate food intake—whether through social learning (e.g., observing peer’s eating behaviors) or modeling (e.g., adopting behaviors from caregivers)—to inform effective obesity prevention strategies in ECE settings. Future EAH assessments in classrooms could explore the impact of offering children different types of snacks. Initiatives to develop ECE curricula that contain techniques to effectively teach hunger and fullness cues during mealtimes should be implemented in combination with culturally competent strategies to prioritize minority populations. Stronger efforts are needed to prevent exposure to and the overconsumption of sweet snacks in early childhood to prevent the life course sequelae of obesity.

## 5. Conclusions

Present findings suggest that a preference for sweet food is associated with EAH, and eating sweet snacks in the absence of hunger is related to higher BMI percentiles among preschool-aged children. Since the frequent snacking of sugary foods and drinks has been associated with BMI among two- to five-year-olds, it is important to highlight that sugary snacks might have a greater influence on BMI than salty snacks. Further, obesity prevention programs may focus on addressing eating sugary foods in the absence of hunger foods starting at an early preschool age.

## Figures and Tables

**Table 1:** Child and parent characteristics (*n* = 211).

Variable	*n* (%)	*M* (*SD*)
**Child characteristics**		
**Sex**		
Male	102 (48%)	-
Female	109 (52%)	-
**Age (months)**		51.5 (5.5)
**ECE facility type**		
Head Start	96 (46%)	-
Childcare facility	115 (54%)	-
**Weight (kg)**	-	18.2 (3.5)
**BMI percentile**		63.0 (28.6)
**Weight status category**		
Normal weight (<85th percentile)	152 (72%)	-
Overweight (85th–<95th percentile)	28 (13%)	-
Obese (≥95th percentile)	31 (15%)	-
**Ethnicity**		
Hispanic	143 (68%)	-
Non-Hispanic	68 (32%)	-
**Yearly household income** ^a^		
USD 0 to USD 24,000	69 (46%)	-
USD 24,001 to USD 39,000	43 (29%)	-
USD 39,001 to USD 78,000	27 (18%)	-
USD 78,001 or more	11 (7%)	-

Note. ^a^ Based on responses from *n* = 150 parent participants who answered the household income item.

**Table 2:** Amount of sweet, salty, and total snacks consumed (in grams) by child sex, ECE facility type, ethnicity, and weight status.

	Sweet Snacks Consumed			Salty Snacks Consumed			Total Snacks Consumed		
Grouping Variable	*M*	*SD*	*p*	*M*	*SD*	*p*	*M*	*SD*	*p*
**Sex**			0.015			<0.001			<0.001
Male	8.01	8.14		5.08	6.17		13.08	9.75	
Female	5.02	5.94		3.00	3.29		8.02	7.25	
**ECE facility type**			0.442			0.170			0.230
Childcare	6.89	6.17		4.41	5.04		11.29	8.80	
Head Start	5.96	7.30		3.52	4.93		9.48	8.96	
**Ethnicity**			0.091			0.102			0.093
Hispanic	5.79	6.73		3.59	4.63		9.38	8.24	
Non-Hispanic	7.88	8.04		4.87	5.62		12.76	9.81	
**Weight status category**			0.745			0.743			0.850
Normal weight	6.31	6.86	0.818	4.12	5.14	0.537	10.43	8.55	0.973
Overweight	7.39	8.04		4.33	5.49		11.72	10.57	
Obese	6.39	8.35		3.16	3.68		9.55	9.13	

Note. *p*-values from comparisons performed in single-covariate generalized estimating equation analyses.

**Table 3:** Coefficients, standard errors, and Wald test statistics from generalized estimating equations relating snack consumption to child’s BMI percentile, adjusting for background covariates (*n* = 211).

	Model 1				Model 2				Model 3			
Predictor	*b*	*SE*(*b*)	Wald	*p*	*b*	*SE*(*b*)	Wald	*p*	*b*	*SE*(*b*)	Wald	*p*
ECE facility type ^a^	5.29	4.43	1.43	0.232	5.54	4.50	1.52	0.218	5.39	4.61	1.37	0.242
Child race/ethnicity ^b^	0.53	3.72	0.02	0.887	0.24	3.76	0.00	0.949	0.61	3.64	0.03	0.866
Child sex ^c^	−1.45	3.77	0.15	0.700	−1.40	3.79	0.14	0.712	−0.77	4.13	0.03	0.852
Preference for sweet snacks	–	–	–	–	−0.77	2.78	0.08	0.782	−1.85	3.11	0.35	0.552
Preference for salty snacks	–	–	–	–	1.96	3.62	0.29	0.589	2.39	3.62	0.43	0.510
Sweet snacks eaten (g)	–	–	–	–	–	–	–	–	0.61	0.27	4.95	0.026
Salty snacks eaten (g)	–	–	–	–	–	–	–	–	−0.57	0.35	2.62	0.106
Wald test of relative model fit	-				Model 2 vs. Model 1 χ^2^ (*df* = 2) = 0.30, *p* = 0.860				Model 3 vs. Model 2 χ^2^ (*df* = 2) = 11.02, *p* = 0.004			

Note. ^a^ Childcare facility is the reference category. ^b^ Non-Hispanic is the reference category. ^c^ Male is the reference category.

## Data Availability

The original contributions presented in this study are included in the article. Further inquiries can be directed to the corresponding author(s).
